# Anti-apoC-III Therapies and Implications for Treatment of Pancreatitis and Cardiovascular Disease

**DOI:** 10.1007/s11883-025-01345-4

**Published:** 2025-10-13

**Authors:** Sotirios Tsimikas

**Affiliations:** Vascular Medicine Program, Sulpizio Cardiovascular Center, University of California San Diego, 9500 Gilman Drive, BSB 1080, La Jolla, CA 92093-0682 USA

**Keywords:** Atherosclerotic cardiovascular disease, Pancreatitis, Apolipoprotein-III, *APOC3*, Hypertriglyceridemia, Familial chylomicronaemia syndrome

## Abstract

**Purpose of Review:**

Apolipoprotein C-III (apoC-III) is a central regulator of triglyceride metabolism. Elevated triglyceride levels are associated with increased risk of acute pancreatitis and atherosclerotic cardiovascular disease (ASCVD).

**Recent Findings:**

Conventional triglyceride-lowering therapies, such as fibrates and omega-3 fatty acids, have limited efficacy in reducing triglycerides and in reducing the risk of pancreatitis or ASCVD in patients with severe hypertriglyceridemia. ApoC-III inhibits lipoprotein lipase and impairs clearance of both triglyceride-rich and cholesterol-rich lipoproteins. Novel therapies targeting *APOC3* mRNA to reduce triglycerides, including antisense oligonucleotides (ASOs) and small interfering RNAs (siRNAs), achieve greater triglyceride reductions than standard agents. Volanesorsen and olezarsen, both ASOs, are approved as an adjunct to diet to reduce triglycerides in familial chylomicronemia syndrome (FCS) in different regions. Plozasiran, an siRNA, is in late-stage clinical development. Indirect cross-trial comparisons were performed, aligned by timepoint and outcome measures, and indicate comparable efficacy of olezarsen and plozasiran in patients with chylomicronemia.

**Summary:**

*APOC3* inhibition is now an established therapeutic approach for reducing the risk of acute pancreatitis in FCS, with three agents, volanesorsen, olezarsen, and plozasiran, demonstrating efficacy. However, its role in ASCVD prevention remains unproven. This review evaluates current *APOC3 *– targeted therapies for FCS, including available comparative data, and synthesizes the emerging literature on the potential of *APOC3* inhibition to reduce the burden of acute pancreatitis in broader populations with severe hypertriglyceridemia, as well as its potential role in ASCVD risk reduction.

## Introduction

Hypertriglyceridemia is a spectrum disorder defined by plasma triglyceride levels ≥ 150 mg/dL, ranging from mild elevations to extreme levels exceeding 10,000 mg/dL. Clinically relevant categories include familial chylomicronemia syndrome (FCS) with estimate prevalence of 1 to 13 per million individuals [1], severe (≥ 500 mg/dL) hypertriglyceridemia (SHTG) prevalent in approximately 1–2% of the U.S. population [2] and mild hypertriglyceridemia (150–499 mg/dL) prevalent in up to 25% of the US population [3, 4]. Higher risk categories within the SHTG population include patients with triglyceride levels ≥ 880 mg/dL and patients with triglyceride levels ≥ 500 mg/dL with a prior history of pancreatitis [5–7]. FCS and SHTG are associated with recurrent acute pancreatitis with risk generally increasing in parallel with triglyceride levels, although substantial interindividual variability exists. In contrast, mild hypertriglyceridemia are linked to increased risk of atherosclerotic cardiovascular disease (ASCVD) [8–10].

Accumulating evidence from preclinical studies, epidemiologic studies, Mendelian randomization analyses and clinical trials support a causal association of triglycerides, triglyceride-rich lipoproteins (TRLs), and TRL remnants with increased risk for pancreatitis and ASCVD [11]. Compared to other forms of acute pancreatitis, triglyceride-related acute pancreatitis is associated with a higher mortality rate, increased incidence of pancreatic necrosis, greater demand for intensive care unit resources, and longer duration of hospitalization [12, 13]. Due to their elevated triglyceride levels, patients with FCS or SHTG experience a significant risk of life-threatening triglyceride-related acute pancreatitis [14].

Conventional triglyceride-lowering drugs reduce apolipoprotein C-III (apoC-III) and triglyceride levels by 10%−30% and 20%−40%, respectively [9]. ApoC-III inhibits lipoprotein lipase (LPL) [15] and hepatic lipase, and delays clearance of TRLs playing a central role in the metabolism of TRLs [16]. Targeted reduction of apoC-III may achieve potent triglyceride lowering and reduce the risk of triglyceride-mediated acute pancreatitis and potentially ASCVD, as suggested in experimental models [17].

This review highlights current and emerging apoC-III–targeted therapies, analyzes and presents comparable efficacy data for olezarsen and plozasiran, evaluates the potential of these therapies to reduce the risk of acute pancreatitis and ASCVD, and summarizes available safety data.

### ApoC-III as a Target to Reduce Plasma Triglycerides

ApoC-III is an 8.8 kDa glycoprotein composed of 79 amino acids, synthesized primarily in the liver and intestine [18]. It is found on TRLs, including chylomicrons, very low-density lipoprotein (VLDL), and their remnants, as well as on high-density lipoprotein (HDL), apoB-100–containing particles, and lipoprotein(a) [Lp(a)] [19]. ApoC-III regulates plasma triglyceride levels and is associated with elevated plasma triglyceride levels and prolonged the circulation of atherogenic lipoproteins.

Genetic studies of loss-of-function (LOF) variants in *APOC3* support a causal role of apoC-III in ASCVD. Heterozygous carriers of the *R19X* null variant exhibit approximately 50% lower apoC-III levels, reduced fasting and postprandial triglyceride concentrations, and a decreased risk of coronary artery calcification compared to noncarriers [20]. These individuals also display a favorable overall lipid profile, which may also contribute to ASCVD risk reduction. This includes lower VLDL-C and remnant cholesterol (remnant-C), higher HDL-C, and an accelerated conversion of VLDL to LDL without changes in direct VLDL clearance [20, 21].

Two large cohort studies published in 2014 confirmed the cardioprotective effects of *APOC3* LOF variants [22, 23]. In the NHLBI Exome Sequencing Project (*n* = 110,970), carriers had 46% lower apoC-III, 39% lower triglyceride levels, and a 40% reduced risk of coronary heart disease compared to noncarriers [22]. Similarly, in the Copenhagen City Heart Study and Copenhagen General Population Study (combined *n* = 75,725), *APOC3* LOF carriers had a 44% reduction in triglyceride levels, along with 41% and 36% reductions in the risks of ischemic vascular disease and ischemic heart disease, respectively [23].

A subsequent meta-analysis suggested that the reduced risk of ischemic vascular disease in *APOC3* LOF carriers is mediated primarily by reductions in remnant-C rather than LDL-C [24]. Since plasma apoC-III levels strongly correlate with both triglycerides and remnant-C, targeting apoC-III represents a promising strategy for reducing ASCVD risk [24]. In fact, in the recent Essence-TIMI 73b study, placebo-adjusted reductions in remnant-C were 66.1% with olezarsen 50 mg and 68.4% with olezarsen 80 mg [25].

### Therapies to Target APOC3

ASOs are synthetic, single-stranded nucleic acid polymers that bind target RNA via Watson-Crick base pairing, leading to RNase H1–mediated degradation primarily in the nucleus and reduced protein synthesis [26]. siRNAs are double-stranded and therefore larger in molecular weight; they harness the endogenous RNA interference pathway, triggering mRNA degradation through the RNA-induced silencing complex in the cytoplasm.

To enhance hepatic delivery, critical since apoC-III is predominantly synthesized in the liver, both ASOs and siRNAs can be conjugated to N-acetylgalactosamine (GalNAc) [27, 28]. GalNAc binds to the asialoglycoprotein receptor on hepatocytes, enabling receptor-mediated endocytosis. Once internalized, the conjugate is processed in endosomes, releasing the oligonucleotide into the cytoplasm. Targeting hepatocytes via GalNAc-conjugated oligonucleotides significantly improves the potency and durability of apoC-III suppression and downstream triglyceride reduction.

## Unexpected Efficacy of APOC3 Inhibition in LPL–Deficient States

### Through Previously Unrecognized LPL-Independent Mechanisms

Anti-*APOC3* therapies were not initially expected to be effective to treat FCS, a condition characterized by complete or near-complete absence LPL activity. Since apoC-III was traditionally thought to exert its effects through inhibition of LPL-mediated hydrolysis of TRLs [29], targeting apoC-III was presumed to be ineffective in the absence of functional LPL.

However, a clinical study involving three patients with genetically confirmed FCS demonstrated that antisense inhibition of APOC3 with volanesorsen led to marked reductions in plasma triglyceride levels [30]. This unexpected result suggested that apoC-III contributes to hypertriglyceridemia through additional mechanisms beyond LPL inhibition, including impaired hepatic clearance of remnant particles demonstrated subsequently [16] and enhancement of VLDL secretion [31]. This revealed an unanticipated biological pathway and broadened the potential therapeutic scope of apoC-III–targeted interventions.

### Targeting ApoC-III for Pancreatitis Reduction

**Fig. 1:**
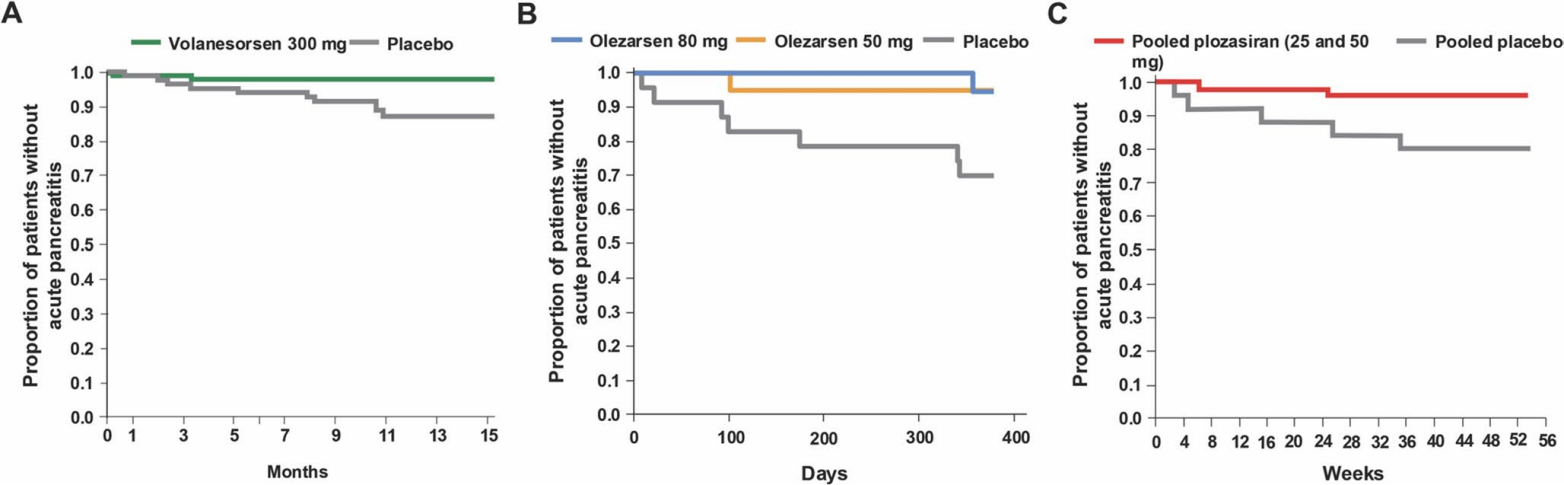
Incidence of acute pancreatitis in patients with severe hypertriglyceridemia treated with volanesorsen (A), olezarsen (B), and plozasiran (C). (**a**) Re-adapted from a meta-analysis of the APPROACH, COMPASS, and BROADEN trials [32] (**b**). Re-adapted from the Balance trial [33]. (**c**) Adapted from the PALISADE trial with permission [34]

SHTG is widely recognized by clinicians and regulatory agencies as a major risk factor for acute pancreatitis. Although several triglyceride-lowering therapies have been approved for patients with triglyceride levels above 500 mg/dL, no reduction in pancreatitis incidence had been demonstrated until 2024 and 2025 when data with RNA directed therapeutics volanesorsen, olezarsen and plozasiran were published [32–34] (Fig. 1).

Volanesorsen is an ASO that targets *APOC3* mRNA to suppress apoC-III production. Unlike newer agents, it is not conjugated to GalNAc and is distributed systemically and may suppress apoC-III production in both the liver and intestine, the 2 largest sources of apoC-III production [35]. Volanesorsen was evaluated in an open-label study involving three patients with FCS who lacked functional LPL activity [30]. Subcutaneous administration once weekly for 13 weeks resulted in substantial reductions in apoC-III (71%–90%) and triglyceride levels (56%–86%). These findings demonstrated that apoC-III modulates triglyceride metabolism through an LPL-independent pathway.

The Phase 3 APPROACH trial evaluated the efficacy and safety of subcutaneous volanesorsen (300 mg once weekly) in 66 patients with FCS over 52 weeks [36]. At 3 months, volanesorsen reduced plasma apoC-III and triglyceride levels by 84.2% and 76.5%, respectively, compared to increases of 6.1% and 17.6% in the placebo group. During the study, acute pancreatitis occurred in three placebo-treated patients with four episodes, versus one volanesorsen treated patient with one episode (occurring nine days after the final dose). Notably, no patient with a prior history of acute pancreatitis in the five years preceding volanesorsen treatment experienced a recurrence.

Volanesorsen was further evaluated in the Phase 3 COMPASS trial, which enrolled 113 patients with SHTG or FCS over 26 weeks [37]. Five adjudicated episodes of acute pancreatitis occurred in the placebo group, while no episodes were reported in the volanesorsen group.

In the BROADEN study, volanesorsen was investigated in patients with familial partial lipodystrophy (FPLD), a rare genetic disorder characterized by selective loss of peripheral subcutaneous adipose tissue, resulting in hypertriglyceridemia, insulin resistance, and increased risk of recurrent pancreatitis [38]. In the randomized phase, three adjudicated episodes of acute pancreatitis occurred in the placebo group, while 4 episodes in 1 patient was reported in the volanesorsen group [32].

Data from the three volanesorsen trials were pooled in a meta-analysis of 207 participants, showing that acute pancreatitis occurred in 2 patients (2%) in the volanesorsen group and 9 patients (10%) in the placebo group during the randomized treatment period (odds ratio, 0.18; 95% CI, 0.04–0.82) [32]. Notably, 10 of the 11 patients who developed pancreatitis during the study had experienced prior episodes before randomization. Kaplan–Meier analysis demonstrated a significant difference in pancreatitis-free survival between treatment groups (Fig. 1A). Trial-specific results were directionally consistent with the meta-analysis findings. Importantly, no cases of acute pancreatitis occurred in the volanesorsen group beyond four months after treatment initiation, coinciding with maximal triglyceride reduction, whereas events continued throughout the study period in the placebo group.

In the recent 2025 focused update of the 2019 ESC/EAS guidelines for the management of dyslipidemias, it was recommended that volanesorsen (300 mg/week) should be considered in patients with severe hypertriglyceridemia (>750 mg/dL or >8.5 mmol/L) due to genetically defined FCS to lower triglyceride levels and reduce the risk of pancreatitis, Class IIA, level of evidence B [39].

#### Olezarsen

Olezarsen is a triantennary GalNAc-conjugated ASO that targets *APOC3* mRNA in the liver, leading to its degradation via RNase H1–mediated cleavage and subsequent reduction in apoC-III production [40]. It shares the same nucleotide sequence as volanesorsen but differs by the addition of the GalNAc3 moiety and its linker, which enables targeted hepatic delivery and localized action, allowing for lower dosing and injection volume.

The Phase 3 BALANCE trial evaluated subcutaneous olezarsen administered every 4 weeks in 66 patients with FCS, most of whom had a history of pancreatitis [33]. After 6 months, olezarsen 80 mg achieved least-squares means placebo-corrected percentage point reductions of 73.7% in apoC-III and 43.5% in triglycerides from baseline. After 12 months, olezarsen 80 mg achieved a least-squares mean placebo- corrected percentage point reduction in triglycerides of 59.4%. In the FDA analysis (https://www.accessdata.fda.gov/drugsatfda_docs/label/2024/218614s000lbl.pdf?utm_source=chatgpt.com), when the reduction in triglycerides for the 80 mg dose was calculated with a placebo-washout imputation, the mean % treatment difference in triglycerides at 6 months was 42.5%. At 12 months, the mean % treatment difference in triglycerides was 57% (unpublished data).

Notably, acute pancreatitis was markedly reduced: only 1 of 22 patients receiving olezarsen 80 mg experienced a single episode (on day 357), compared to 11 episodes in 7 of 23 placebo-treated patients, beginning as early as day 9 (Fig. 1B). In a secondary endpoint, pooled patients treated with olezarsen 80 or 50 mg had a numerically lower incidence of acute pancreatitis compared with placebo (mean rate ratio, 0.12).

Suspected acute pancreatitis events were adjudicated by a blinded, independent clinical research organization using a committee of pancreatitis experts and the revised Atlanta classification and definitions by international consensus [41]. Because patients with recurrent pancreatitis are not always evaluated with the full complement of diagnostic tests and clinical diagnosis is common, the committee also assigned a graded level of evidence for events adjudicated as positive. Each event was classified as documented, probable, possible, unable to adjudicate, or no acute pancreatitis. Documented acute pancreatitis required at least two of the following: [1] abdominal pain strongly consistent with acute pancreatitis (acute onset of persistent, severe epigastric pain often radiating to the back); [2] serum lipase or amylase ≥ 3× the upper limit of normal; [3] characteristic findings on contrast-enhanced CT, MRI, or transabdominal ultrasonography. In BALANCE, 13 events were adjudicated as positive for acute pancreatitis with levels of evidence of 12 documented and 1 possible (Table 1).

**Table 1:** Details of adjudicated cases of pancreatitis in the balance trial based on revised Atlanta classification classification and definitions by international consensus

Treatment arm	Atlanta Criteria Classification
Placebo	Documented
Placebo	Documented
Placebo	Documented
Placebo	Documented
Placebo	Documented
Placebo	Documented
Placebo	Documented
Placebo	Possible
Placebo	Documented
Placebo	Documented
Placebo	Documented
Olezarsen 50 mg	Documented
Olezarsen 80 mg	Documented

In 2024, olezarsen was approved by the FDA as an adjunct to diet for reducing triglyceride levels in adults with FCS. Importantly, genetic confirmation of FCS was not required for eligibility. Olezarsen is currently being investigated for its efficacy and safety in patients with SHTG (Essence-TIMI 73b; NCT05610280 [42] CORE-TIMI 72a; NCT05079919 [43], CORE2-TIMI 72b NCT05552326 [43].

#### Plozasiran

Plozasiran is a triantennary GalNAc3-conjugated siRNA that suppresses hepatic production and secretion of apoC-III [34, 44]. The Phase 3 PALISADE trial evaluated the efficacy and safety of subcutaneous plozasiran administered every 3 months in 75 patients with persistent chylomicronemia, including both genetically confirmed FCS and patients with symptomatic persistent chylomicronemia suggestive of FCS.

After 10 months, plozasiran 50 mg reduced placebo-adjusted median triglyceride and apoC-III levels by 53% and 93%, respectively. The 25 mg dose achieved comparable reductions of 59% and 91%, respectively [34]. In a pooled analysis, plozasiran also significantly reduced the incidence and delayed the onset of acute pancreatitis: 2 events occurred in 2 of 50 (4%) in the 25 mg plozasiran group, compared with 7 events in 5 of 25 (20%) placebo group (Fig. 1C).

### Indirect Comparison of Olezarsen and Plozasiran on ApoC-III and Triglyceride Levels

Although no head-to-head trials have compared olezarsen and plozasiran, indirect comparisons, despite differences in study design and populations, can provide insight into their relative efficacy. Table 2 summarizes the key design features of the Balance [33] and PALISADE [34] trials. The Balance study enrolled patients with genetically confirmed FCS, with centralized adjudication of genetic causality in a core laboratory. In contrast, the PALISADE trial included both genetically confirmed FCS or presumed FCS, without noting the method of genetic determination or whether the testing was centralized or adjudicated. Furthermore, the PALISADE study reported median triglyceride values as the primary summary statistical analysis for triglyceride outcomes, versus mean levels in Balance. The Palisade analysis was placebo-adjusted by design (via the “median difference” estimand), but the reporting of the results does not always clearly reflect this.

**Table 2:** Key design features of the balance and PALISADE trials

Design Feature	Balance	PALISADE
Therapy Studied	Olezarsen (ASO)	Plozasiran (siRNA)
Target Population	FCS	Genetically confirmed 59%) or presumed FCS ((41%)*
Genetic Confirmation	Required	Not required
Genetic Testing Method	Central core laboratory adjudication	Not described
Inclusion TG Threshold	TG ≥ 880 mg/dL	TG ≥ 880 mg/dL
Sample Size	66 participants	75 participants
Duration of Follow-up	1 year	1 year
Primary Endpoint Reporting	Placebo-adjusted mean % change in TG	Placebo-adjusted median % change in TG
Primary Endpoint Timepoint	Month 6	Month 10
Statistical Measures of Variability	95% confidence intervals (CIs)	Interquartile ranges (IQRs)

*The original protocol was designed to evaluate only patients who had FCS with an established genetic diagnosis, but the protocol was amended to include patients with symptomatic, persistent chylomicronemia suggestive of FCS [34]

To enhance comparability, placebo-adjusted median values from the olezarsen 80 mg and plozasiran 25 mg in PALISADE were aligned at similar timepoints (Table 3). ApoC-III reductions were nearly identical: − 87% at week 53 for olezarsen (95% CI: − 112, − 69) and − 87% at 10 months for plozasiran (95% CI: − 113, − 61). Triglyceride reductions were also similar, though numerically greater with plozasiran: − 60% (–92, − 28) vs. − 49% (–77, − 21) with olezarsen. These findings suggest comparable efficacy in lowering apoC-III and triglycerides, although formal statistical comparison is not possible due to lack of primary data for PALISADE, the presence of two populations of study subjects in PALISADE versus only genetically diagnosed FCS in Balance and methodological differences in data analysis and reporting. The most appropriate comparison would be to analyze change at similar timepoints in placebo-corrected mean or median values of apoC-III and triglycerides in the genetically confirmed FCS subjects in both studies.

**Table 3:** Comparison of changes in median (95% CI) apoC-III and triglyceride levels in the balance and PALISADE trials

Placebo-adjusted median percent change from baseline [1] (95% CI)	Olezarsen (Balance) 80 mg	Plozasiran (PALISADE) 25 mg
Time point	Week 53	Month 12 [2]
ApoC-III, mg/dL	−87 (− 112, − 69)	−87 (− 113, − 61)
Time point	Mean of Weeks 51 and 53 (i.e., Month 12)	Mean of Months 10 and 12
Triglyceride [3,4], mg/dL	−49 (− 77, − 21)	−60 (− 92, − 28)

Olezarsen 95% CI of treatment difference was calculated using Hodges-Lehmann estimate. Olezarsen values were not previously published and were derived from additional analyses from the Balance trial [33]. PALISADE [34] data was publicly available. ApoC-III, apolipoprotein C-III; CI, confidence interval

[1] Baseline was defined as the average of the pre-dose measurement on Day 1 and the last non-missing measurement closest to Day 1 (last measurements from Qualification period and Screening period), prior to administration of the first dose of Study Drug

[2] Month 12 was defined as the average of Weeks 51 and 53. If 1 assessment was missing, then the other non-missing assessment was used

[3] For triglyceride, missing data after last dosing date for patients in the Olezarsen arms were imputed using washout MI (J2R) approach. Missing data before the last dosing date or for patients in the placebo group were imputed under the MAR assumption

[4] For triglyceride, Hodges-Lehmann estimate of the location shift with 95% CI were the overall estimates combined from all 100 tests

Despite similar reductions in apoC-III, numerical differences in triglyceride lowering between groups is hypothesized to reflect differences in LPL activity in the study populations, although neither study measured LPL activity. For example, in the APPROACH trial of FCS, which enrolled subjects with both genetically confirmed and clinically confirmed FCS, similar reductions in apoC-III with achieved with volanesorsen as expected based on its mechanism. Both groups were able to achieve significant triglyceride reductions, but the triglyceride responses were modestly attenuated in the genetically diagnosed patients, suggesting that clinically diagnosed patients may recruit residual LPL activity that leads to more potent triglyceride reduction [45]. Consistent with this observation, in patients with moderate hypertriglyceridemia (triglyceride levels 150–500 mg/dL) where LPL activity is presumed to be normal, two phase 2 studies of olezarsen effects [46, 47] and the recent Essence-TIMI 73b trial showed robust triglyceride lowering. In Essence-TIMI 73b encompassing 1349 patients with mild hypertriglyceridemia (150–499 mg/dL), at 6 months the placebo-adjusted least-squares mean change in triglyceride level was − 58.4% points (95% confidence interval [CI], − 65.1 to − 51.7; *P* < 0.001) in the olezarsen 50-mg group and − 60.6% points (95% CI, − 67.1 to − 54.0; *P* < 0.001) in the olezarsen 80-mg group [25].

### Targeting APOC3 To Reduce ASCVD

In individuals with triglyceride levels between 150 and 500 mg/dL, often seen in insulin resistance, metabolic syndrome, or type 2 diabetes, elevated apoC-III promotes accumulation of atherogenic remnant lipoproteins that are causally linked to ASCVD. Even modest triglyceride elevations are associated with subclinical atherosclerosis and vascular inflammation, particularly in statin-treated patients with normal LDL-C levels [4, 9].

Conventional triglyceride-lowering therapies—such as fibrates, niacin, and omega-3 fatty acids—modestly reduce apoC-III levels, limiting their efficacy in reducing ASCVD risk [18]. In contrast, therapies targeting *APOC3* achieve greater reductions in triglycerides, remnant cholesterol, and TRLs, though they exert modest effects on LDL-C and apoB compared to statins or PCSK9 inhibitors. As such, they may be most effective when combined with LDL-C–lowering agents to address residual cardiovascular risk.

While APOC3 inhibition improves many lipid parameters, interpreting its impact on ASCVD risk is complicated by apparent increases in LDL-C, especially in individuals with impaired TRL clearance, such as those with FCS or severe hypertriglyceridemia. In these patients, baseline LDL-C is often artificially low due to defective VLDL remodeling [32, 33, 35, 45, 46]. As apoC-III is suppressed and TRL clearance improves, LDL particles may reappear or increase, reflecting a normalization of lipoprotein metabolism rather than a true rise in atherogenic burden [30, 33, 34, 36, 48]. Importantly, non–HDL-C and apoB levels usually decline modestly, supporting a net benefit. LDL-C changes with *APOC3*-targeted therapies should therefore be interpreted alongside broader markers such as apoB and remnant cholesterol.

Whether these favorable lipid changes translate into cardiovascular risk reduction remains an open question. Large outcome trials have yielded mixed results. PROMINENT and STRENGTH, which tested fibrates and high-dose omega-3 fatty acids respectively, failed to show ASCVD risk reduction despite lowering triglycerides [49, 50]. In contrast, REDUCE-IT demonstrated a 25% reduction in ischemic events with icosapent ethyl, despite only a modest 19.7% median triglyceride reduction—suggesting pleiotropic mechanisms beyond lipid lowering and/or adverse effects of the mineral oil placebo [51].

Notably, the apoC-III reductions achieved in STRENGTH (7.0%) and PROMINENT (27.8%) were substantially smaller than those observed with *APOC3* loss-of-function mutations [49, 50], possibly explaining the lack of benefit. More potent suppression of apoC-III, as achieved with ASO and siRNA therapies, may be necessary to impact cardiovascular outcomes meaningfully.

### Effects of APOC3 Inhibitors on Atherogenic Lipoproteins

Table 4 summarizes the effects of APOC3 inhibitors on atherogenic lipoproteins. ASO and siRNA therapies targeting apoC-III have shown consistent and robust reductions in TRLs, along with favorable changes in HDL-C and other atherogenic markers across multiple clinical trials.

**Table 4:** Effect of volanesorsen, olezarsen, and Plozasiran on lipid and lipoprotein profiles in patients with hypertriglyceridemia (≥ 150 mg/dL)

Studies	ApoC-III	TG	LDL-C	VLDL-C	HDL-C	Non-HDL-C	ApoB
Volanesorsen							
Phase 2, Gaudet et al. [48]							
Cohort A: monotherapy (TG ≥ 350 and ≤ 2000 mg/dL)^a^	↓39–80%	↓31–71%	↑47–111%	↓40–70%	↑28–46%	↓6.3–12%	↑1.5–7.1%
Cohort B: added to fibrate (TG ≥ 225 and ≤ 2000 mg/dL)^a^	↓61–72%	↓52–65%	↓2.4–↑19%	↓55–64%	↑48–49%	↓18–20%	↓10–13%
Phase 2, Digenio et al. [52]							
Type 2 diabetes and TG > 200 and < 500 mg/dL)^b^	↓88%	↓69%	0.00%	↓73%	↑43%	↓22%	↓21%
Olezarsen							
Phase 2 Tardif et al. [46]							
ASCVD or moderate triglycerides (TG ≥ 200 and ≤ 500 mg/dL)^c^	↓30–74%	↓27–63%	↓1.0–↑23%	↓25–57%	↑12–42%	↓7.0–24%	↓16–↑2%
Bridge Phase 2b Bergmark et al. [47]							
Moderate hypertriglyceridemia (TG ≥ 150 and < 500 mg/dL) and elevated CV risk or TG ≥ 500 mg/dL)^d^	↓64–73%	↓49–53%	↓7.7%–9.9%	↓46%–50%	↑40%	↓23–25%	↓18–19%
Essence-TIMI 73b, Phase 3, Bergmark et al. [25]	↓62–70%	↓58–61%	↓1.1%–1.5%	↓57%–57%	↑46–48%	↓22–22%	↓15–15%
Moderate hypertriglyceridemia (TG 150–500 mg/dL) and elevated cardiovascular risk or severe hypertriglyceridemia (TG ≥ 500 mg/dL)^c^							
Plozasiran							
MUIR Phase 2b [53]							
Mixed hyperlipidemia (TGs ≥ 150 and < 500 mg/dL)^d^	↓56–79%	↓44–62%	↓14%–↑3%		↑28–46%	↓7.7–24.2%	↓6.5–19.1%

^a^Values represent the least-squares mean % change from baseline

^b^Values represent the mean % change from baseline

^c^Values represent the least-squares mean difference in % change from baseline compared with placebo

^d^Values represent the least-squares mean difference in % change from baseline compared with placebo (percentage points)

ApoA-I, apolipoprotein A-I; ApoB, apolipoprotein B; ApoC-III, apolipoprotein C-III; ASCVD, atherosclerotic cardiovascular disease; HDL-C, high-density lipoprotein cholesterol; triglycerides, hypertriglyceridemia; LDL-C, low-density lipoprotein cholesterol; REM-C, remnant cholesterol; TG, triglycerides; VLDL-C, very low-density lipoprotein cholesterol

Volanesorsen demonstrated potent lipid-modifying effects in patients with hypertriglyceridemia. In a Phase 2 study [48], 13 weeks of treatment with volanesorsen 300 mg resulted in statistically significant mean percent change reductions in VLDL-C (–69.2%) and apoB-48 (–61.1%), along with a 45.7% increase in HDL-C compared to placebo. Although increases were observed in LDL-C and LDL-apoB, levels of non–HDL-C and total apoB remained unchanged. Volanesorsen also reduced the size of chylomicron–VLDL particles, suggesting enhanced clearance of atherogenic TRLs. Additionally, the drug lowered apoC-III levels across multiple lipoprotein classes, including apoB-containing particles, Lp(a), and apoA-I–containing HDL [19].

Olezarsen has demonstrated robust efficacy in reducing TRLs and atherogenic markers in high-risk populations. In a Phase 2 study involving patients with ASCVD or moderate hypertriglyceridemia with 80–90% on background statin therapy, olezarsen significantly reduced non–HDL-C, total apoB, and VLDL-C, while increasing HDL-C without affecting LDL-C levels [46]. Further analysis in a nuclear magnetic resonance study showed a 51% reduction in total TRL levels, a favorable shift in LDL particle size (186% increase in large LDL, 39% decrease in small LDL), and increased HDL particle counts [54]. Notably, 91% of patients treated with olezarsen 50 mg achieved triglyceride levels < 150 mg/dL, and 45% reached < 100 mg/dL [46]. Similar results were observed in the Bridge trial, where olezarsen 80 mg lowered triglycerides to < 150 mg/dL in over 93% of treated patients, compared to just 11.8% of those receiving placebo [47]. In the Essence-TIMI 73b study [25] changes in levels of apolipoprotein C-III, statistically significant placebo-adjusted reductions in both olezarsen doses were noted in VLDL-C (−57%), non-HDL-C (−22%), directly measured remnant cholesterol (−62 to 70%), apolipoprotein B (−15%), with no significant change in LDL-C (−1.1 to −1.5%).

Plozasiran demonstrated significant lipid-modifying effects in the Phase 2b MUIR trial [53]. Treatment increased HDL-C and reduced remnant-C, non–HDL-C, and total apoB. Up to 92% of patients receiving 25 or 50 mg quarterly achieved triglyceride levels < 150 mg/dL, compared to 21% in the placebo group. The reduction in non–HDL-C was primarily driven by decreases in remnant-cholesterol rather than LDL-C. In a recent meta-analysis in non-FCS patients [55], olezarsen and plozasiran both significantly reduced triglycerides, apoC-III, and non-HDL-C, and increased HDL-C. However, olezarsen did not significantly increase LDL-C, while plozasiran was associated with dose-dependent increases in LDL-C, particularly at higher doses. Whether these differences will be noted in phase 3 trials or impact hard CV outcomes remains unknown and requires dedicated cardiovascular outcomes trials to determine whether these lipid improvements translate into reduced events.

To evaluate the cardiovascular effects of apoC-III inhibition, an Essence-TIMI 73b trial substudy [25] is evaluating whether olezarsen-mediated reductions in apoC-III and triglycerides influence coronary plaque progression or stabilization using coronary CT angiography.

**Fig. 2:**
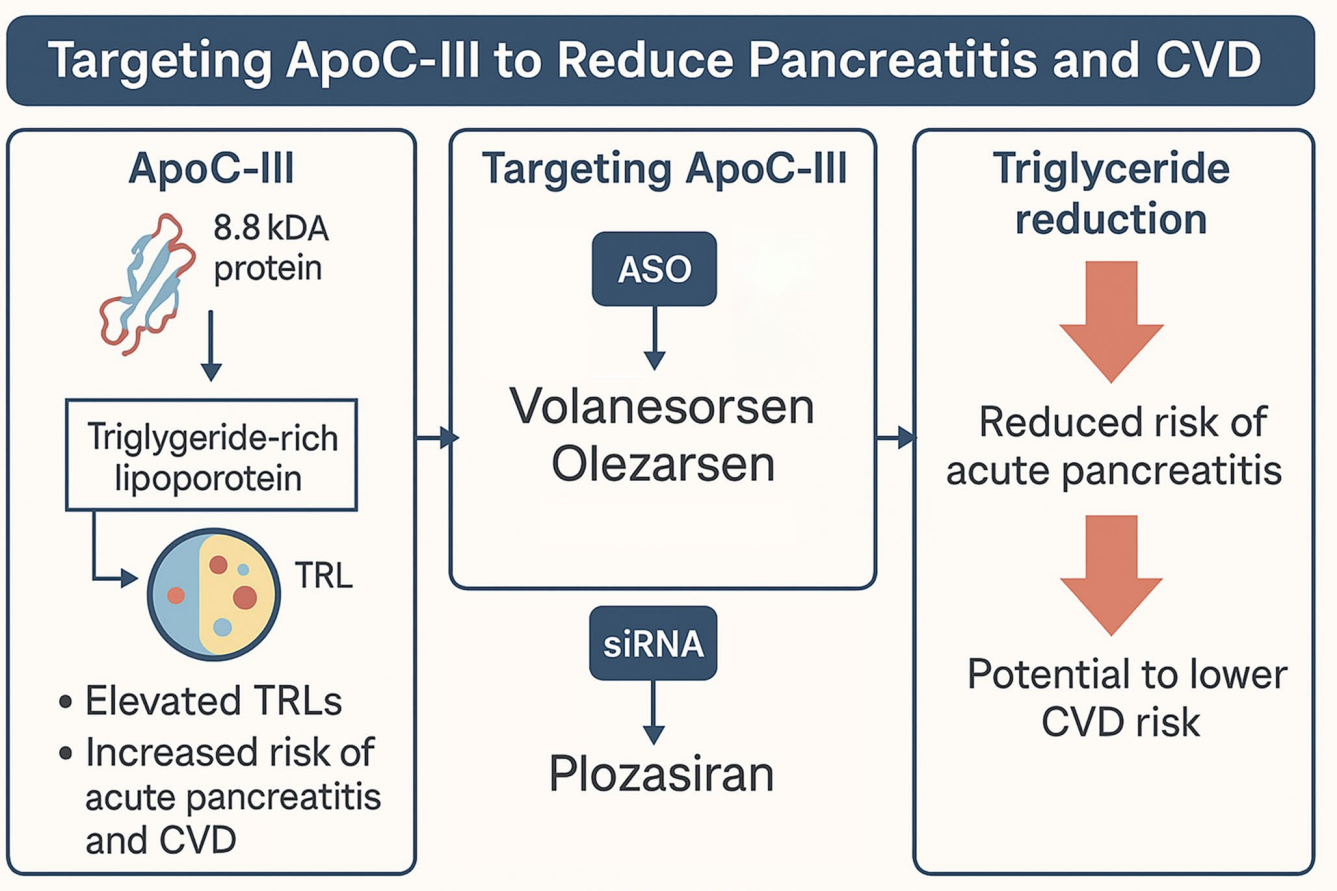
Apolipoprotein C-III (apoC-III) regulates triglyceride metabolism by inhibiting lipolysis and remnant clearance, contributing to elevated triglyceride-rich lipoproteins (TRLs) and increased risk of acute pancreatitis and cardiovascular disease (CVD). Therapies targeting apoC-III, such as volanesorsen, olezarsen, and plozasiran, use ASO or siRNA technology to reduce apoC-III production and significantly lower triglyceride levels. These treatments have demonstrated reduced risk of acute pancreatitis and may potentially lower CVD risk, pending further clinical outcomes data

The effect of apoC-III in regulating triglyceride metabolism by inhibiting lipolysis and remnant clearance, and therapies targeting it—such as volanesorsen, olezarsen, and plozasiran—to lower triglycerides and reduce pancreatitis risk, with potential cardiovascular benefits is summarized in Fig. 2.

### Safety and Tolerability

Nucleic acid therapies targeting APOC3 mRNA have generally reassuring safety profiles; however, administration-related adverse events (AEs) are reported across individual agents and the class. Injection-site reactions, most of which are characterized as mild, which are among the most common AEs across volanesorsen, olezarsen, and plozasiran trials. Hypersensitivity reactions have been also reported in patients treated with nucleic acid therapies. In non-GalNAc-modified volanesorsen, reversible decreases in platelet count were present. For GalNAc-modified ASOs, generally mild and transient declines in platelet counts have been noted, but these have not led to clinically meaningful bleeding or cessation of therapy. Overall, the proportion of patients experiencing a bleeding adverse event was similar across the olezarsen and placebo treatment groups. No clinically relevant changes have been noted in renal function.

Some patients with diabetes or prediabetes experienced worsening glycemic control during treatment with plozasiran [34, 53, 56]. However, assessments using the homeostatic model assessment of insulin resistance (HOMA-IR) did not indicate significant changes in insulin sensitivity [53, 56]. The underlying mechanisms for the observed deterioration in glycemic control are not yet elucidated [34, 53, 56]. The COMPASS trial in patients with SHTG with volanesorsen, but not APPROACH in FCS or BROADEN in FPLD, reported worsened glycemic control in patients with diabetes treated with volanesorsen [37]. In the olezarsen studies, no glucose abnormalities were reported in the Phase 1 study of healthy volunteers with mild hypertriglyceridemia [40]. In the Phase 2 Bridge trial, no statistically significant differences were present in HbA1c at 6 or 12 months although variability was present [47]. In Balance, small increases in average values in fasting glucose (≤ 17 mg/dL) and HbA1c (< 0.2% points) were observed over time with olezarsen treatment in the FCS population (olezarsen/tryngolza FDA label). In Essence-TIMI 73b, increases in glycated hemoglobin levels by absolute 0.3% at 6 months as observed among patients with a diagnosis of diabetes mellitus, but not in those without diabetes. There was no change in HOMA-IR or beta cell function with olezarsen as compared with placebo in patients with or without diabetes.

Dyslipidemia and elevated triglyceride levels are closely associated with non-alcoholic fatty liver disease (NAFLD). Significant reductions in apoC-III and triglycerides could theoretically promote hepatic steatosis by increasing peripheral and hepatic uptake of free fatty acids and TRLs, although further data in this area is required as the current data are inconsistent. For example, an analysis of the APPROACH, COMPASS, and BROADEN trials found that volanesorsen treatment led to a ~ 20–50% reduction in hepatic fat fraction compared with placebo in patients with various causes of hypertriglyceridemia [57]. In these studies, liver biopsies to assess inflammation and fibrosis were not performed. In a prespecified exploratory substudy of SHASTA-2 [56], hepatic fat fraction was assessed via MRI-PDFF in a substudy of 49 participants (17 placebo; 9, 12, and 11 receiving plozasiran 10 mg, 25 mg, and 50 mg, respectively). Baseline liver fat ranged from 17.9% to 22.4% across groups. At week 24, least-squares mean changes in hepatic fat were − 0.4% for placebo, −0.3% for 10 mg, 0.0% for 25 mg, and + 2.3% for 50 mg, leading to placebo-adjusted values of −0.7%, 0.4% and 2.5% for the 3 doses, respectively. The effect of olezarsen on hepatic fat fraction was not assessed in Balance, Bridge or Essence-TIMI 73b, but is currently under investigation in CORE and CORE2 studies in individuals with triglycerides >500 mg/dL.

## Conclusions

Epidemiologic, clinical, and genetic evidence strongly support apoC-III as a therapeutic target for reducing the risk of pancreatitis. Advances in genetic and molecular technologies have led to the development of *APOC3*-mRNA targeted therapies that achieve substantial reductions in apoC-III and triglyceride levels, far exceeding those achieved with conventional lipid-lowering agents. Clinical trials of volanesorsen, olezarsen, and plozasiran have demonstrated clear benefits in lowering pancreatitis risk in the highest risk patients with FCS or presumed FCS. Ongoing studies of SHTG patients are awaited to expand these findings in broader populations. A role of apoC-III inhibition in ASCVD is suggested by a modest reduction in non-HDL and apoB, but no results from imaging or outcomes studies have yet been published. Continued research will be critical to determining how reductions in apoC-III, triglycerides, and atherogenic lipoproteins may translate into cardiovascular risk reduction.

### Key References

- Gordts PL, Nock R, Son NH, Ramms B, Lew I, Gonzales JC, et al. ApoC-III inhibits clearance of triglyceride-rich lipoproteins through LDL family receptors. J Clin Invest. 2016;126 (8):2855-66. https://www.ncbi.nlm.nih.gov/pubmed/27400128.
  - This study documents the clearance pathways affected by apoC-III and provides a rationale for triglyceride lowering by inhibition of *APOC3* mRNA with a mechanism independent of lipoprotein lipase.
- Pollin TI, Damcott CM, Shen H, Ott SH, Shelton J, Horenstein RB, et al. A null mutation in human APOC3 confers a favorable plasma lipid profile and apparent cardioprotection. Science. 2008;322(5908):1702-5. https://www.ncbi.nlm.nih.gov/pubmed/19074352.
  - This study demonstrates that loss of function mutations were associated with lower fasting and postprandial serum triglycerides, lower levels of LDL-C and subclinical atherosclerosis, suggesting that lifelong deficiency of apoC-III has a cardioprotective effect.
- Bergmark BA, Marston NA, Prohaska TA, Alexander VJ, Zimerman A, Moura FA, et al. Targeting APOC3 with Olezarsen in Moderate Hypertriglyceridemia. N Engl J Med. 2025;0(0). https://www.ncbi.nlm.nih.gov/pubmed/40888739.
  - This study demonstrates significant reductions in apoC-III, triglycerides and atherogenic lipoproteins, including VLDL-C, non-HDL-C, and apoB.
- Gaudet D, Brisson D, Tremblay K, Alexander VJ, Singleton W, Hughes SG, et al. Targeting APOC3 in the familial chylomicronemia syndrome. N Engl J Med. 2014;371 (23):2200-6. https://www.ncbi.nlm.nih.gov/pubmed/25470695.
  - This study supports the role of APOC3 as a key regulator of LPL-independent pathways of triglyceride metabolism.
- Alexander VJ, Karwatowska-Prokopczuk E, Prohaska TA, Li L, Geary RS, Gouni-Berthold I, et al. Volanesorsen to prevent acute pancreatitis in hypertriglyceridemia. N Engl J Med. 2024;390 (5):476-7. https://www.ncbi.nlm.nih.gov/pubmed/38294982.
  - This is the first documentation, using a meta-analysis approach, in the literature that acute pancreatitis events could be reduced by a pharmacological agent, volanesorsen.
- Stroes ESG, Alexander VJ, Karwatowska-Prokopczuk E, Hegele RA, Arca M, Ballantyne CM, et al. Olezarsen, acute pancreatitis, and familial chylomicronemia syndrome. N Engl J Med. 2024;390 (19):1781-92. https://www.ncbi.nlm.nih.gov/pubmed/38587247.
  - In patients with familial chylomicronemia syndrome, olezarsen may represent a new therapy to reduce plasma triglyceride levels.
- Watts GF, Rosenson RS, Hegele RA, Goldberg IJ, Gallo A, Mertens A, et al. Plozasiran for managing persistent chylomicronemia and pancreatitis risk. N Engl J Med. 2025;392 (2):127 − 37. https://www.ncbi.nlm.nih.gov/pubmed/39225259.
  - Patients with FCS or presumed FCS who received plozasiran had significantly lower triglyceride levels and a lower incidence of pancreatitis than those who received placebo.
- Witztum JL, Gaudet D, Freedman SD, Alexander VJ, Digenio A, Williams KR, et al. Volanesorsen and triglyceride levels in familial chylomicronemia syndrome. N Engl J Med. 2019;381 (6):531 − 42. https://www.ncbi.nlm.nih.gov/pubmed/31390500.
  - Volanesorsen lowered triglyceride levels to less than 750 mg per deciliter in 77% of patients with familial chylomicronemia syndrome.
- Tsimikas S, Ginsberg HN, Alexander VJ, Karwatowska-Prokopczuk E, Dibble A, Li L, et al. Differential effects of volanesorsen on apoC-III, triglycerides, and pancreatitis in familial chylomicronemia syndrome diagnosed by genetic or nongenetic criteria. J Clin Lipidol. 2025;19 (3):422 − 31.
  - For a similar reduction in apoC-III in response to volanesorsen, triglyceride reduction is attenuated in patients with genetically vs. nongenetically diagnosed FCS.

## Data Availability

Access to the data may be granted on reasonable request to qualified researchers with appropriate regulatory and institutional review board authorization approvals. Requests for data will be evaluated based on scientific merit, feasibility, and alignment with the original study objectives.
